# Dr. Herman Reeve Ainsworth

**Published:** 1914-11

**Authors:** 


Dr. Herman Reeve Ainsworth, N. Y. University, 1866, died at his home in Addison, N. Y., September 3. Beside being a member of the A. M. A. and affiliated societies, he was a member of the American Assn. for the Advancement of Science, of the Keuka Medical Society and the Greater N. Y. Medical Association.

